# Abnormal Vibration Signal Detection of EMU Motor Bearings Based on VMD and Deep Learning

**DOI:** 10.3390/s25185733

**Published:** 2025-09-14

**Authors:** Yanjie Cui, Weijiao Zhang, Zhongkai Wang

**Affiliations:** 1 Postgraduate Department, China Academy of Railway Sciences, Beijing 100081, China; cuiyjhhh@163.com; 2 Institute of Computing Technology, China Academy of Railway Sciences Corporation Limited, Beijing 100081, China; winter-light@163.com

**Keywords:** EMU motor bearings, anomaly detection, time series prediction, VMD, OC-SVM

## Abstract

To address the challenge of anomaly detection in vibration signals from high-speed electric multiple unit (EMU) motor bearings, characterized by strong non-stationarity and multi-component coupling, this study proposes a synergistic approach integrating variational mode decomposition (VMD) and deep learning. Unlike datasets focused on fault diagnosis (identifying known fault types), anomaly detection identifies deviations into unknown states. The method utilizes real-world, non-real-time vibration data from ground monitoring systems to detect anomalies from early signs to significant deviations. Firstly, adaptive VMD parameter selection, guided by power spectral density (PSD), optimizes the number of modes and penalty factors to overcome mode mixing and bandwidth constraints. Secondly, a hybrid deep learning model integrates convolutional neural networks (CNNs), bidirectional long- and short-term memory (BiLSTM), and residual network (ResNet), enabling precise modal component prediction and signal reconstruction through multi-scale feature extraction and temporal modeling. Finally, the root mean square (RMS) features of prediction errors from normal operational data train a one-class support vector machine (OC-SVM), establishing a normal-state decision boundary for anomaly identification. Validation using CR400AF EMU motor bearing data demonstrates exceptional performance: under normal conditions, root mean square error (RMSE=0.005), Mean Absolute Error (MAE=0.002), and Coefficient of Determination $\left(R^{2}=0.999\right)$; for anomaly detection, accuracy = 95.2% and F1-score = 0.909, significantly outperforming traditional methods like Isolation Forest (F1-score = 0.389). This provides a reliable technical solution for intelligent operation and maintenance of EMU motor bearings in complex conditions.

## 1. Introduction

Bearings are critical components in EMUs, whose performance directly affects the operational safety and reliability of trains. In recent years, with the continuous expansion of EMU fleet size and increased operational frequency, the load and working intensity borne by bearings have significantly increased. According to fault statistics reported by Song et al. [1] over five consecutive years, bearing failures in the transmission system are primarily distributed in axle box bearings and gearbox bearings, which together account for more than 90% of all faults. Although motor bearings account for only 6% of total failures, their abnormal vibrations can still directly impact the stability of the entire transmission system and its operational safety. Therefore, research on anomaly detection in motor bearing vibration signals is of great importance for early fault diagnosis and prevention. Analyzing non-real-time vibration data collected by ground monitoring systems can help identify potential anomalies, providing an effective means for early fault detection and condition trend assessment [2,3]. However, in engineering practice, anomaly samples representing typical fault states are extremely scarce or even entirely absent [4,5]. In this context, unsupervised anomaly detection methods based solely on normal operational data have become a crucial technical approach for achieving early fault identification in bearings.

Hiruta et al. [6] propose an unsupervised learning method based on Gaussian Mixture Models. By grouping power spectral features and estimating model parameters using the Expectation-Maximization algorithm, it achieves detection of abnormal conditions in motor bearings. Evaluation via area under the curve (AUC) demonstrates the method’s effectiveness in distinguishing between normal and abnormal states. Several references [7,8,9] have employed support vector machine techniques, combined with various signal processing and feature extraction methods, to achieve high-accuracy rolling bearing fault diagnosis. Other works [10,11,12,13] have focused on autoencoder-based methods. For example, Shen et al. [10] proposed a dynamically loss-guided autoencoder (AE) to suppress randomness and drift in feature extraction, thus improving diagnostic performance. Diez et al. [11] demonstrated that autoencoders outperform traditional principal component analysis (PCA) in detecting fully rotating and oscillating bearings, with superior sensitivity, accuracy, and recognition capabilities of early degradation. Dai et al. [12] propose a dual-path self-supervised learning-based method for bearing anomaly detection. This approach achieves precise early detection of weak faults across operating conditions using only normal vibration data by capturing global features through contrastive learning and extracting local details via a reconstruction mechanism. Kang et al. [13] propose a dual-input deep anomaly detection method that achieves early fault warning for rolling bearings using only normal vibration data through the integration of dual-channel high-frequency signal inputs and an experience replay mechanism. Liu et al. [14] combined autoencoders with Wasserstein generative adversarial networks (WGANs) to efficiently detect faults in unlabeled data. König et al. [15] pioneer an unsupervised approach using autoencoders to detect sliding bearing anomalies. Trained solely on normal-condition acoustic-emission features, the model identifies abnormalities through reconstruction error thresholds. Temporal prediction approaches have also been explored in the literature [16,17]. Lee et al. [16] used CNN to extract spatial features, coupled with BiLSTM networks and regression layers to optimize temporal learning. Xu et al. [17] developed a hybrid model that integrates long- and short-term memory (LSTM) networks, generative adversarial networks (GAN) and extreme gradient boost (XGBoost) to extract deep temporal characteristics and improve the efficiency of rolling bearing fault detection.

Furthermore, recent advancements have integrated VMD with deep learning to enhance feature extraction from noisy and non-stationary vibration signals. Habbouche et al. [18] proposed a VMD-based notch filter to cancel the dominant mode in vibration signals, combined with a 1D-CNN, achieving high-accuracy bearing fault detection and diagnosis under noisy conditions on the CWRU dataset. Xiong et al. [19] developed an optimized VMD using an enhanced Sparrow Search Algorithm (OCSSA) for parameter selection and integrated it with a CNN-BiLSTM network, significantly improving the diagnosis accuracy of rolling bearings under strong noise interference. Qiu et al. [20] introduced a War Strategy Optimization (WSO) algorithm to adaptively optimize VMD parameters and proposed a ResNet-SWIN model, effectively enhancing the diagnosis capability for weak faults in rotation vector reducer crankshaft bearings under variable-speed conditions.

In the EMU domain, Ding [21] proposes an impact-response-based convolutional sparse coding method that effectively detects anomalies by resolving nonlinear and non-stationary modulation issues in wheelset bearings using test-rig data. Deng et al. [22] enhance detection accuracy for high-speed train axle bearings through Singular Value Decomposition (SVD) denoising combined with Adaptive Time-Reassignment Multisynchrosqueezing Transformer, utilizing operational parameters such as temperature and speed. For real-time anomaly detection, Jin et al. [23] develop Time Density-Weighted Incremental Support Vector Data Description to improve the timeliness and accuracy of online roller bearing monitoring; Liu et al. [24] construct a hybrid multi-layer LSTM and Isolation Forest model that reduces early warning response latency for high-speed EMU axle box bearings through temperature time-series prediction and deviation-index dynamic monitoring.

**Figure 1:**
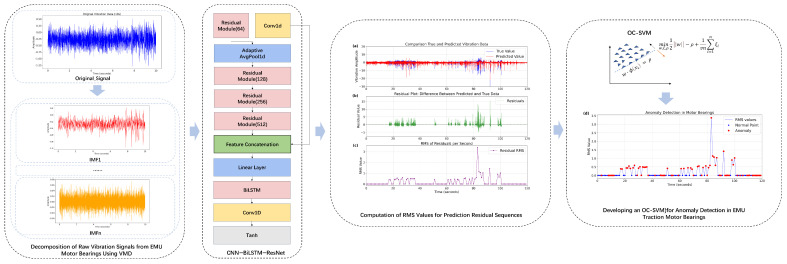
Anomaly detection framework for EMU motor bearings based on VMD-CBR-OCSVM anomaly detection model.

This study proposes an innovative anomaly detection method for the non-real-time vibration signals of the motor bearings in the CR400AF high-speed train. The proposed VMD-CBR-OCSVM model establishes a cohesive analytical pipeline. First, the raw vibration signal is fed into the VMD module, which adaptively decomposes the complex signal into a series of IMFs through power-spectrum-guided parameter optimization. These IMFs are then processed by a hybrid CNN-BiLSTM-ResNet (CBR) network for temporal prediction. By leveraging the combined strengths of convolution, bidirectional long short-term memory, and residual connections, the CBR network accurately forecasts the future values of each component and reconstructs the full signal by summing them. Subsequently, the residual between the predicted and original signals is computed, and its RMS value is derived as a discriminative feature representing state deviations. Finally, this RMS feature is input into OC-SVM, where unsupervised anomaly identification is achieved by comparing it against the decision boundary learned solely from normal data. The research framework is illustrated in Figure 1.

Unlike previous research that relies on laboratory simulations or publicly available datasets, this integrated framework is validated using data collected from real operational environments. This ensures both a large dataset and high sampling frequency, while more accurately capturing the complexity and variability of actual operating conditions—providing essential, realistic data for training each stage of the proposed model. Laboratory simulation data are typically idealized, making it difficult to account for random factors and subtle faults present in real-world environments, while publicly available datasets often suffer from issues such as insufficient sample size and inadequate sampling frequency. By utilizing this unique dataset to validate a complete, end-to-end detection framework, this study not only fills a gap in this field but also provides solid technical support for the application of deep learning in anomaly detection for EMUs. The proposed VMD-CBR-OCSVM framework constitutes an integrated analytical pipeline, wherein each algorithmic component is meticulously engineered to address a specific challenge inherent in the unsupervised anomaly detection of non-stationary vibration signals. The functional roles and synergistic interactions of these components are delineated as follows:

- VMD functions as an adaptive signal preprocessor. Its primary role is to mitigate the analytical challenges posed by strong non-stationarity and multi-component coupling in the raw vibration signals. By leveraging a power spectrum-guided mechanism to autonomously optimize its key parameters, VMD decomposes the complex source signal into a finite set of band-limited, quasi-orthogonal IMFs. This decomposition effectively disentangles the complex signal into simpler, more structured sub-components, thereby enhancing the signal-to-noise ratio and providing a refined input for the subsequent temporal modeling stage.
- The CBR hybrid network serves as the core spatio-temporal predictor. This module is designed to learn the complex underlying patterns representative of normal bearing operation. The architecture synergistically combines the strengths of its constituent deep learning models: the CNN layers extract salient local spatial features and patterns from the input IMFs; the BiLSTM layers capture long-range temporal dependencies and contextual information both forwards and backwards in time; and the ResNet components, through skip connections, facilitate the training of a deeper network by alleviating the vanishing gradient problem and enabling robust feature fusion. The objective of this network is to learn a high-fidelity predictive model of normal system dynamics. Consequently, any significant deviation from the expected behavior, manifested as a substantial prediction error, serves as a potent indicator of an emerging anomaly.
- The OC-SVM operates as the final unsupervised anomaly detector. This component is tasked with interpreting the prediction residuals generated by the CBR network. The RMS value of the prediction error sequence is calculated as a discriminative feature, chosen for its sensitivity to changes in signal energy. The OC-SVM is trained exclusively on the RMS values derived from normal operation data. Utilizing a Radial Basis Function (RBF) kernel, it learns a tight decision boundary that encapsulates the intrinsic distribution of normal condition features. Any subsequent observation that falls outside this learned boundary is classified as an anomaly, thus enabling fully unsupervised detection without any requirement for labeled fault data.

This integrated framework establishes a coherent workflow tailored for EMU motor bearing monitoring, combining adaptive signal decomposition, temporal modeling and statistical decision-making to form a robust solution for early fault detection in high-speed railway traction systems. The main contributions of this study are as follows:

- A novel adaptive VMD preprocessing method: We introduce a power spectrum-guided mechanism that autonomously optimizes the critical decomposition parameters (mode number and penalty factor). This physics-informed approach eliminates the reliance on empirical tuning, effectively addressing the challenges of strong non-stationarity and low signal-to-noise ratio in raw vibration signals, thereby ensuring robust and reliable signal decomposition.
- An advanced spatio-temporal prediction network: We design a hybrid CBR architecture as a core predictor. This network innovatively combines convolutional layers for spatial feature extraction, bidirectional LSTM layers for capturing long-range temporal dependencies, and residual connections to facilitate the training of a deep network and enable effective feature fusion. This design allows for high-fidelity modeling of the complex dynamics inherent in normal bearing vibration signals.
- A practical unsupervised detection framework: We establish a complete anomaly-detection pipeline that requires only normal operational data for training. By using the RMS of the prediction errors from the CBR network as a sensitive feature—selected for its proven effectiveness as a stable time-domain statistic that is highly sensitive to changes in signal energy caused by bearing faults [25,26,27]—and training an OC-SVM model, the framework learns the intrinsic distribution of normal conditions. This approach provides a viable and practical solution for real-world applications where labeled fault data are scarce or unavailable.

## 2. Power-Spectrum-Guided Variational Mode Decomposition Parameter Optimization

VMD is a variational optimization-based adaptive signal decomposition method with significant application value in non-stationary signal processing. High-speed-train motor bearing vibration signals are characterized by strong non-stationarity, multi-component coupling, and low signal-to-noise ratio. Traditional Empirical Mode Decomposition (EMD) methods exhibit inherent limitations, notably mode mixing, under these conditions. In contrast, VMD mitigates the noise sensitivity inherent in recursive decomposition via its frequency-domain energy-adaptive partition mechanism. The decomposition dimension is governed by the number of modes *K*, while the bandwidth penalty factor α regulates the bandwidth of the extracted modes, establishing a dual-parameter optimization framework. Simultaneously, the iterative update strategy for the center frequencies effectively prevents spectral overlap. This combination provides a robust analytical approach for signal decoupling under complex operating conditions.

### 2.1. Determination of the Number of Modes

Within the VMD algorithm, the number of decomposition modes, denoted as *K*, determines the quantity of IMFs generated. Each IMF represents the frequency characteristics inherent in the original vibration signal. For performance degradation analysis of high-speed-train motor bearings using VMD, the selection of parameters *K* and α is critical. An appropriate *K* value ensures accurate feature extraction, avoiding both over-decomposition and under-decomposition. Similarly, a suitable α value facilitates accelerated convergence and enhanced noise resistance, thus enabling the effective detection of motor bearing abnormalities. Consequently, optimizing these two parameters significantly improves the accuracy of motor bearing anomaly detection under non-real-time conditions.

Significant peaks in the PSD typically reveal the main components of the signal. If the PSD, calculated using the Welch method [28], displays multiple distinct peaks, it suggests that the signal may contain multiple different vibration sources. In such cases, selecting too small a value for “*K*” may result in mode mixing, where different vibration sources are incorrectly decomposed into the same IMF. Conversely, if the PSD shows fewer peaks, selecting an excessively large “*K*” may lead to redundant decomposition, generating unnecessary IMFs that often only contain noise or secondary components. By analyzing the distribution characteristics of the PSD, an appropriate value for “*K*” can be chosen, ensuring sufficient decomposition of the signal while avoiding noise interference. The optimization procedure for the modal count *K* comprises the following steps:

Step 1: Define the original signal. Assume that the vibration signal from the motor bearings of EMU, after denoising and smoothing, is s(t). This is a continuous signal in the time domain. However, in practical processing, it is typically represented by a discrete sampled signal. Given the sampling rate $f_{s}$, the sampling interval is $\Delta t=1/f_{s}$ and the number of sampling points is *N*. The discrete signal can be expressed as:

(1) s(n)=s(nΔt),n=0,1,2,…,N−1

Step 2: Compute the power spectral density using Welch’s method.

(2) $$P_{x}\left(f\right)=\frac{1}{N_{s}f_{s}}\sum_{m=0}^{M-1}{\left|\sum_{n=0}^{N_{s}-1}s_{m}\left(n\right)\omega \left(n\right)e^{-j2\pi fn/f_{s}}\right|}^{2}$$

In this context, $P_{x}\left(f\right)$ denotes the PSD; *f* represents the frequency vector defined over $\left[0,f_{s}/2\right]$; $s_{m}\left(n\right)$ corresponds to the *m*-th signal segment with length $N_{s}$; ω(n) signifies the window function of length $N_{s}$; *M* indicates the total number of signal segments; and $N_{s}$ specifies the length of each signal segment.

Step 3: Detect significant peaks. The peak detection formula is defined as:

(3) $$P_{x}\left(f_{p}\right)>\theta \cdot \max\left(P_{x}\left(f\right)\right)$$

where θ is the threshold coefficient; $f_{p}$ denotes the peak frequency satisfying the condition; $N_{p}$ represents the number of qualified peak frequencies; and the initial number of modes $K_{i}$ is estimated as:

(4) $$K_{i}=N_{p}+1$$

Define the candidate range $K_{c}$:

(5) $$K_{c}=\left[\max\left(2,K_{i}-2\right),K_{i}+2\right]$$

Step 3: Compute modal separability and reconstruction error. For every *K* within the candidate range, decompose the signal into *K* modes ${\left\{u_{k}\left(t\right)\right\}}_{k=1}^{K}$, with the optimization objective formulated as:

(6) $$\min_{\left\{u_{k},\omega_{k}\right\}}\left\{\sum_{k=1}^{K}{∥\partial_{t}\left[\left(u_{k}\left(t\right)+\frac{j}{\pi t}\right)*\delta \left(t\right)e^{-i\omega_{k}t}\right]∥}_{2}^{2}\right\}$$

subject to the constraint:

(7) $$\sum_{k=1}^{K}u_{k}\left(t\right)=s\left(t\right)$$

where δ(t) denotes the unit impulse function, *j* is the imaginary unit, ∗ represents the convolution operator, $\partial_{t}$ signifies the time derivative, $\omega_{k}$ is the center frequency of the *k*-th IMF, and ${∥\cdot ∥}_{2}$ indicates the $L^{2}$ norm. To solve this constrained optimization problem, we introduce the Lagrange multiplier λ(t) and penalty factor α, constructing the augmented Lagrangian function as follows:

(8) $$\begin{array}{cc} L(\left\{u_{k}\right\},\left\{\omega_{k}\right\},\lambda ) & =\alpha \sum_{k}{∥\partial_{t}\left[\left(u_{k}\left(t\right)+\frac{j}{\pi t}\right)*\delta \left(t\right)e^{-i\omega_{k}t}\right]∥}_{2}^{2}\\ \; & \;+{∥f\left(t\right)-\sum_{k}u_{k}\left(t\right)∥}_{2}^{2}\\ \; & \;+\langle \lambda \left(t\right),f\left(t\right)-\sum_{k}u_{k}\left(t\right)\rangle \end{array}$$

where 〈·,·〉 denotes the inner product. The modes $u_{k}$ and center frequencies $\omega_{k}$ are iteratively updated and finally output as their discrete representations $u_{k}\left(n\right)$ and $\omega_{k}$. For each mode $u_{k}\left(n\right)$, its center frequency is computed as:

(9) $$f_{c}^{\left(k\right)}=\frac{\sum_{f}f\cdot P_{x}^{\left(k\right)}\left(f\right)}{\sum_{f}P_{x}^{\left(k\right)}\left(f\right)}$$

where $P_{x}^{\left(k\right)}\left(f\right)$ is the power spectral density of $u_{k}\left(n\right)$. The modal separability $S_{K}$ is defined as the minimum interval between adjacent center frequencies; a larger $S_{K}$ indicates less mode mixing.

(10) $$S_{K}=\min_{i\neq j}\left|f_{c}^{\left(i\right)}-f_{c}^{\left(j\right)}\right|,\;i=1,2,\ldots ,K$$

If K=1, then $S_{K}=0$. The reconstructed signal is:

(11) $$\hat{s}\left(n\right)=\sum_{k=1}^{K}u_{k}\left(n\right)$$

The reconstruction error $R_{K}$ is defined as the normalized mean square error between the original signal and the reconstructed signal; the smaller $R_{K}$, the more complete the original information preserved by the modal components.

(12) $$R_{K}=\frac{\sum_{n=0}^{N-1}{\left(s\left(n\right)-\hat{s}\left(n\right)\right)}^{2}}{\sum_{n=0}^{N-1}s{\left(n\right)}^{2}}$$

Step 5: Optimize the objective function to obtain the optimal number of decomposition modes. Select the optimal *K* for EMU motor bearing signals, defining the objective function as:

(13) $$Q_{K}=S_{K}-\lambda_{c}\cdot R_{K}$$

where $\lambda_{c}$ is the weight coefficient. Optimize the objective function to balance separability and error, preserving information of the original vibration signal without over-decomposition or under-decomposition. The optimal number of decomposition modes $K_{p}$ is:

(14) $$K_{p}=\arg\max_{K\in K_{c}}Q_{K}$$

### 2.2. Determination Method for the Penalty Factor

In EMU motor bearing anomaly detection, the bandwidth of the power spectral density reflects the frequency dispersion degree of the signal. If the power spectral density exhibits broadband distribution, it indicates that the signal contains rich frequency components. In this case, a smaller α value is recommended to relax the bandwidth constraints of IMFs, effectively capturing dynamic characteristics of broadband signals. If the power spectral density shows power concentrated in specific narrow frequency bands, potential local fault features may exist. Under such conditions, selecting a larger α value tightens the bandwidth of IMFs, enhancing frequency locality of modes to improve identification accuracy of narrowband fault features. The determination steps for penalty factor α are as follows: In EMU motor bearing anomaly detection, the bandwidth of the power spectral density reflects the frequency dispersion degree of the signal. If the power spectral density exhibits broadband distribution, it indicates that the signal contains rich frequency components. In this case, a smaller α value is recommended to relax the bandwidth constraints of IMFs, effectively capturing dynamic characteristics of broadband signals. If the power spectral density shows power concentrated in specific narrow frequency bands, potential local fault features may exist. Under such conditions, selecting a larger α value tightens the bandwidth of IMFs, enhancing frequency locality of modes to improve identification accuracy of narrowband fault features. The determination steps for penalty factor α are as follows:

Step 1: After determining the optimal number of modes $K_{p}$, systematically adjust the value of α within the test range from 100 to 5000 with a step size of 100 to optimize anomalous signal extraction.

Step 2: For each α value, perform VMD decomposition using Equations (6)–(8). Output $K_{p}$ modes $u_{k}\left(n\right)$.

Step 3: Calculate bandwidth stability. For each mode $u_{k}\left(n\right)$, compute its bandwidth and evaluate stability. First, calculate the power spectral density for each mode:

(15) $$P_{x}^{\left(k\right)}\left(f\right)=\frac{1}{N_{s}f_{s}}\sum_{m=0}^{M-1}{\left|\sum_{n=0}^{N_{s}-1}u_{k}\left(n\right)\omega \left(n\right)e^{-j2\pi fn/f_{s}}\right|}^{2}$$

Then calculate its peak frequency, half-power points, lower/upper cutoff frequencies, and bandwidth using Equations (16)–(20).

(16) $$f_{p}^{\left(k\right)}=\arg\max_{f}P_{x}^{\left(k\right)}\left(f\right)$$

(17) $$P_{h}^{\left(k\right)}=\frac{\max_{f}P_{x}^{\left(k\right)}\left(f\right)}{2}$$

(18) $$f_{l}^{\left(k\right)}=\max\left\{f<f_{p}^{\left(k\right)}|P_{x}^{\left(k\right)}\left(f\right)\leq P_{h}^{\left(k\right)}\right\}$$

(19) $$f_{u}^{\left(k\right)}=\min\left\{f>f_{p}^{\left(k\right)}|P_{x}^{\left(k\right)}\left(f\right)\leq P_{h}^{\left(k\right)}\right\}$$

(20) $$B_{k}=f_{u}^{\left(k\right)}-f_{l}^{\left(k\right)}$$

Finally, compute the bandwidth stability $B_{s}$:

(21) $$B_{s}=\frac{\mathrm{mean}\left({\left\{B_{k}\right\}}_{k=1}^{K_{p}}\right)}{\mathrm{std}\left({\left\{B_{k}\right\}}_{k=1}^{K_{p}}\right)+\epsilon }$$

where $\epsilon =10^{-6}$ prevents division by zero. mean() and std() denote calculating the mean and standard deviation of different bandwidths, respectively. A higher $B_{s}$ value indicates smaller variation in bandwidths $B_{k}$ relative to their mean (i.e., smaller standard deviation). Therefore, a higher $B_{s}$ value signifies better consistency or stability of bandwidths, meaning the bandwidth remains relatively unchanged under different measurement conditions. A lower $B_{s}$ value indicates larger variation in bandwidths $B_{k}$ relative to their mean (i.e., larger standard deviation). This shows significant bandwidth variation across conditions, lacking stability. Thus, a higher $B_{s}$ value indicates better stability of modal bandwidths and more consistent decomposition results. Directly select the α that maximizes $B_{s}$, i.e.,

(22) $$\alpha_{p}=\arg\max_{\alpha }B_{s}\left(\alpha \right)$$

The overall procedure for the power-spectrum-guided variational mode decomposition parameter optimization method is described in Algorithm 1.

**Algorithm 1.** Adaptive VMD parameter optimization.1:**Input**: Discrete vibration signal s(n), sampling rate $f_{s}$, window function ω(n), segment parameters $M,N_{s}$, threshold θ, weight $\lambda_{c}$2:**Output**: Optimal modes $\left\{u_{k}\left(n\right)\right\}$, optimal parameters $K_{p}$, $\alpha_{p}$3:Compute Welch power spectrum density $P_{x}\left(f\right)$ using Equation (2)4:Detect significant peaks: $N_{p}=\sum_{f}1\left[P_{x}\left(f_{p}\right)>\theta \cdot \max\left(P_{x}\left(f\right)\right)\right]$5:

$K_{i}\leftarrow N_{p}+1$

6:Define candidate range $K_{c}=\left[\max\left(2,K_{i}-2\right),K_{i}+2\right]$7:**for** each $K\in K_{c}$ **do**8:        Perform VMD decomposition with current *K* using Equations (6)–(8)9:        Calculate center frequencies $f_{c}^{\left(k\right)}$ using Equation (9)10:        Compute separation $S_{K}=\min_{i\neq j}\left|f_{c}^{\left(i\right)}-f_{c}^{\left(j\right)}\right|$11:        Compute reconstruction $\hat{s}\left(n\right)=\sum_{k=1}^{K}u_{k}\left(n\right)$12:        Calculate error $R_{K}=\frac{\sum {\left(s\left(n\right)-\hat{s}\left(n\right)\right)}^{2}}{\sum s{\left(n\right)}^{2}}$13:        Evaluate $Q_{K}=S_{K}-\lambda_{c}\cdot R_{K}$14:
**end for**
15:

$K_{p}\leftarrow \arg\max_{K\in K_{c}}Q_{K}$

16:Define α range A={100,200,…,5000}17:**for** each α∈A **do**18:        Perform VMD with $K_{p}$ and current α19:        **for** each mode $u_{k}\left(n\right)$ **do**20:                Compute $P_{x}^{\left(k\right)}\left(f\right)$ using Equation (15)21:                Find $f_{p}^{\left(k\right)}$, $f_{l}^{\left(k\right)}$, $f_{u}^{\left(k\right)}$ using Equations (16)–(19)22:                Calculate bandwidth $B_{k}=f_{u}^{\left(k\right)}-f_{l}^{\left(k\right)}$23:        **end for**24:        Compute stability $B_{s}\left(\alpha \right)=\frac{\mathrm{mean}(\left\{B_{k}\right\})}{\mathrm{std}\left(\left\{B_{k}\right\}\right)+10^{-6}}$25:
**end for**
26:

$\alpha_{p}\leftarrow \arg\max_{\alpha \in A}B_{s}\left(\alpha \right)$

27:Perform final VMD with $K_{p}$ and $\alpha_{p}$ to get $\left\{u_{k}\left(n\right)\right\}$28:**Return**$\left\{u_{k}\left(n\right)\right\}$, $K_{p}$, $\alpha_{p}$

## 3. Time-Series Forecasting and Anomaly Detection Based on Deep Learning Networks

### 3.1. IMF Time-Series Forecasting Based on Deep Learning Networks

**Figure 2:**
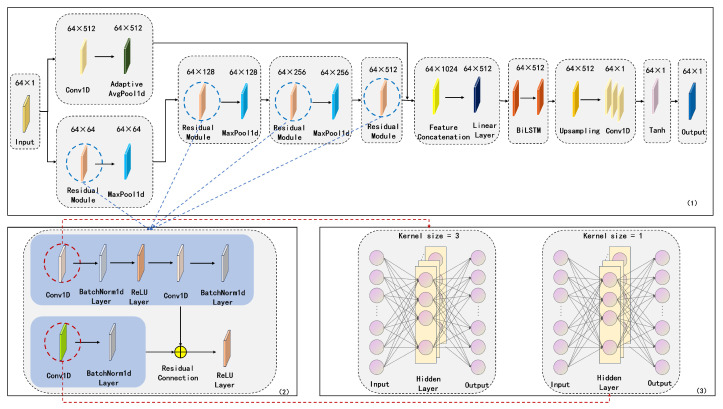
CBR Time-series prediction framework. (1) Overview of the overall architecture. (2) Internal structure of the residual module. (3) One-dimensional convolutional networks with different kernel sizes.

Since motor bearing vibration signals in high-speed train sets typically contain components of different frequencies, processing these signals requires models capable of distinguishing various frequency features. Traditional time-series models often struggle to directly handle such complex signals. By decomposing the signal using the VMD method, K intrinsic mode functions $\left\{{\mathrm{IMF}}_{u_{1}}\left(t\right),\;{\mathrm{IMF}}_{u_{2}}\left(t\right),\;\ldots ,\;{\mathrm{IMF}}_{u_{k}}\left(t\right)\right\}$ are obtained, effectively extracting different frequency components from the signal. To improve prediction accuracy, we employ a deep learning architecture termed CBR, which integrates CNN, BiLSTM, and ResNet. The overall structure of this CBR time-series prediction framework is depicted in Figure 2, and its processing workflow operates as follows:

The input layer of the network receives *K* intrinsic mode functions derived from VMD. First, the signals are processed through a residual convolutional encoding module composed of multiple residual blocks. This module progressively expands the input channels from 1 to 64 via convolutional operations, then increases to 128, further to 256, and finally to a 512-dimensional feature representation. Each residual block incorporates standardized convolution and ReLU activation functions, effectively mitigating the vanishing gradient problem through residual connections. During feature extraction, the temporal dimension is gradually compressed via pooling operations, significantly reducing computational complexity while preserving critical spatial features of the signals.

Subsequently, the network employs a bidirectional long short-term memory network for temporal modeling. This module comprises two BiLSTM layers: the first layer receives 512-dimensional input features and performs bidirectional temporal modeling with 256 hidden units; the second layer further refines the features into 128-dimensional hidden states. By processing sequences bidirectionally (forward and backward), the BiLSTM comprehensively captures long-term dependencies and complex dynamic characteristics in vibration signals, making it particularly suitable for identifying periodic patterns and anomalous evolution trends in motor bearing vibration signals.

During feature propagation, the network introduces an innovative residual connection mechanism. The original input signals undergo channel dimension adjustment via convolutional layers and are then aligned with features from the main path through adaptive pooling, enabling cross-layer feature fusion. This design significantly enhances the retention of original signal characteristics, effectively prevents information degradation in deep networks, and improves model robustness.

Finally, temporal features are mapped to predictions through a fully connected layer module. This module employs a two-stage linear transformation: first reducing the 128-dimensional features to 64 dimensions, then compressing them to a 1-dimensional output. ReLU activation functions are applied between stages to strengthen nonlinear expressive capabilities. The prediction results for each IMF are superimposed to reconstruct the complete vibration signal. By analyzing the deviation patterns between predicted and actual signals, precise monitoring of motor bearing operational states and early fault diagnosis are achieved.

This architecture synergizes CNN’s spatial feature extraction, BiLSTM’s temporal dynamic modeling, and ResNet’s deep optimization mechanisms. It significantly enhances the accuracy and robustness of vibration signal prediction while preserving critical signal characteristics.The detailed hyperparameter configurations and training details are provided in Table 1.

### 3.2. Data Reconstruction and Anomaly Detection

#### 3.2.1. Data Reconstruction

Based on the proposed prediction model, time-series forecasting is performed on the *K* modal functions obtained from VMD, yielding their corresponding predicted values $\left\{\mathrm{IM}{\hat{F}}_{u_{1}}\left(t\right),\;\mathrm{IM}{\hat{F}}_{u_{2}}\left(t\right),\;\ldots ,\;\mathrm{IM}{\hat{F}}_{u_{k}}\left(t\right)\right\}$. The data reconstruction process is achieved by linearly superimposing all predicted $\mathrm{IM}{\hat{F}}_{u_{k}}\left(t\right)$, with the expression given as follows:

(23) $$\hat{s}\left(t\right)=\sum_{i=1}^{K}\mathrm{IM}{\hat{F}}_{u_{k}}\left(t\right)$$

The predicted signal $\hat{s}\left(t\right)$ is obtained by summing all the $\mathrm{IM}{\hat{F}}_{u_{k}}\left(t\right)$ components. This effectively preserves the multi-scale frequency characteristics of the original signal and integrates the dynamic properties of each component, thereby achieving prediction through multi-band information fusion.

#### 3.2.2. Anomaly Detection

Given the practical challenge of acquiring labeled abnormal vibration signals in electric multiple-unit motors, which aligns with the objective of detecting unknown anomalies, this study employs an unsupervised approach by training exclusively on normal signals. This strategy ensures the model learns a robust representation of normal operational patterns without being biased by specific, known faults, thereby enhancing its sensitivity to general deviations. A reserved independent test dataset (containing both normal and abnormal samples) is used to evaluate the generalization performance of the VMD-CNN-BiLSTM-ResNet hybrid model in distinguishing anomalous states. (This process refers to decomposing signals via VMD and applying the CBR time-series prediction framework).

The processing workflow involves first calculating residuals between actual and predicted values of the original vibration signals, then capturing local degradation characteristics, segmenting the residual signals into multiple sliding windows of length $N_{w}$, subsequently computing the RMS value for each window, and ultimately training to establish a binary classifier through the OC-SVM algorithm based on these RMS values, where each window’s RMS value is treated as a single training sample for OC-SVM. During the testing phase, anomaly detection is implemented via this trained binary classifier, where the RMS is calculated as:

(24) $$\mathrm{RMS}=\sqrt{\frac{1}{N_{w}}\sum_{t=1}^{N_{w}}{\left(s\left(t\right)-\hat{s}\left(t\right)\right)}^{2}}$$

This feature quantifies the cumulative deviation of the predictive model in the time domain, effectively characterizing bearing state degradation.

The core of the algorithm lies in solving for an optimal separating hyperplane in the feature space, formulated as the optimization problem:

(25) $$\min_{w,\xi ,\rho }\;\frac{1}{2}{\left|w\right|}^{2}-\rho +\frac{1}{\nu n}\sum_{i=1}^{n}\xi_{i}$$

subject to:

(26) $$\begin{array}{ccc} \; & \left(w\cdot \phi \left(x_{i}\right)\right)\geq \rho -\xi_{i},\;i=1,\ldots ,n\;\; & \xi_{i}\geq 0,\;i=1,\ldots ,n \end{array}$$

Here, *w* represents the normal vector of the hyperplane; $\xi_{i}$ denotes the slack variable; ρ indicates the offset of the hyperplane; *n* signifies the number of training samples; $x_{i}$ corresponds to the RMS feature value of the *i*-th sample; and ϕ(·) represents a nonlinear mapping implemented through the RBF kernel $K\left(x_{i},x_{j}\right)=\exp(-\gamma |x_{i}-x_{j}{|}^{2})$, where $\gamma =1/\mathrm{Var}(X_{train})$ automatically adapts to the data distribution. The hyperparameter ν=0.05, which controls the model’s sensitivity to outliers, was set to 0.05. This value was selected through a cross-validation process designed to minimize false alarms, with the detailed empirical justification provided in Section 4.4.1.

The decision function f(x)=w·ϕ(x)−ρ defines the signed distance in the feature space. The output threshold τ is dynamically determined by the ν-quantile of the decision values from the training data:

(27) $$\tau =\inf\left\{z:P\left(f\left(x\right)\leq z|x\in X_{\mathrm{train}}\right)\geq \nu \right\}$$

The final state determination rule is given by:

(28) $$g\left(x\right)=\left\{\begin{array}{cc} 0\;(\mathrm{Normal}) & \mathrm{if}\;f(x)\geq \tau \\ 1\;(\mathrm{Anomalous}) & \mathrm{if}\;f(x)<\tau \end{array}\right.$$

In this way, it can effectively reduce false positives while maintaining detection sensitivity, thereby enhancing the accuracy and practical applicability of abnormal motor bearing detection in EMUs.

## 4. Experiments and Result Analysis

### 4.1. Data Description

**Figure 3:**
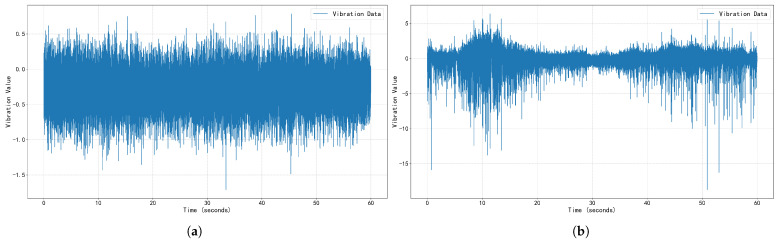
Comparison of normal vibration signal examples versus mixed signal examples containing both normal and abnormal vibrations. (**a**) Normal vibration signal examples. (**b**) Mixed signal examples containing both normal and abnormal vibrations.

This study validates the proposed anomaly detection method using vibration data (sampling frequency: 10 kHz) collected from the same traction motor bearing of a CR400AF EMU during actual commercial operation. Crucially, all abnormal data used for testing represent real operational faults and were not simulated. The data labels (normal/abnormal) were authoritatively derived from the comprehensive diagnostic conclusions of the industrial-grade Prognostics and Health Management (PHM) system developed by the China Academy of Railway Sciences (CARS). This system integrates multi-source information including time-domain, frequency-domain, and temperature analyses, with final determinations made based on expert-defined thresholds and rules. Data from the first three days were classified as normal healthy states for constructing baseline models, while data from the fourth day contained both normal vibration data and confirmed abnormal vibration data. Comparative examples of these vibration states are illustrated in Figure 3 (with a duration of 1 min).

### 4.2. Parameter Optimization for Motor Bearing Vibration Signal Decomposition

**Figure 4:**
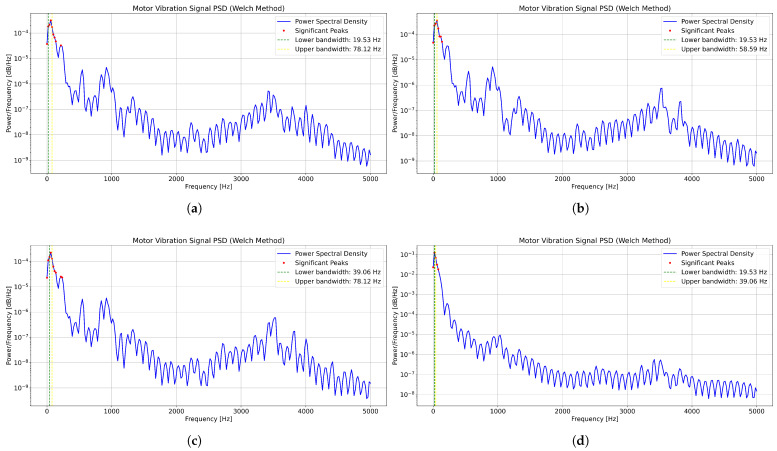
Analysis of PSD for motor bearing vibration signals. (**a**) Day 1 PSD of motor bearing vibration signals. (**b**) Day 2 PSD of motor bearing vibration signals. (**c**) Day 3 PSD of motor bearing vibration signals. (**d**) Day 4 PSD of motor bearing vibration signals.

Based on the VMD method, the vibration signals of motor bearings in EMUs were decomposed using a power-spectrum-guided parameter optimization approach to determine the optimal decomposition mode number *K* and penalty factor α. The PSD of the motor vibration signals, calculated via the Welch method, is shown in Figure 4. PSD provides an estimate of the power distribution across different frequencies, where visible peaks correspond to significant frequency components related to the motor’s operational state and potential fault modes.

The bandwidth, determined by calculating the frequency range where the PSD drops to half of its maximum value (i.e., the −3 dB point), indicates the frequency region containing significant signal energy. This is essential for understanding signal behavior and detecting anomalies. Figure 4 displays the PSD over four consecutive days, reflecting variations in the motor vibration signal under different operational conditions. The significant spectral peaks and the marked bandwidth (with lower and upper frequency bounds) highlight key frequency components and energy concentration ranges, which play a crucial role in the VMD process by supporting the identification of signal modes and potential faults.

By analyzing the PSD results across these four days, the initial range of the decomposition mode number *K* was preliminarily determined according to the prominent spectral peaks observed each day, as summarized in Table 2.

Subsequently, the optimal decomposition mode number $K_{p}$ was determined by optimizing the objective function incorporating modal separability and reconstruction error. After identifying the optimal $K_{p}$, the penalty factor $\alpha_{p}$ was determined by adjusting α values and calculating bandwidth stability. The optimal parameters for different dates are presented in Table 3.

The results in Table 2 and Table 3 demonstrate two key findings: firstly, the initial range for K derived from PSD analysis effectively captures the time-varying spectral complexity of the bearing signals; secondly, the optimized parameters ($K_{p}$, $\alpha_{p}$) are highly sensitive to the signal’s health state. The consistency of parameters on Days 1–3 reflects signal stability under normal conditions, while the significant parameter shift on Day 4 ($K_{p}$ decreases, $\alpha_{p}$ increases) provides a clear, preliminary indicator of anomalous signal behavior characterized by reduced modal complexity and a need for stricter bandwidth control during decomposition. This validates the adaptive optimization mechanism as a crucial step for subsequent accurate prediction and detection.

### 4.3. Prediction Performance Analysis

**Figure 5:**
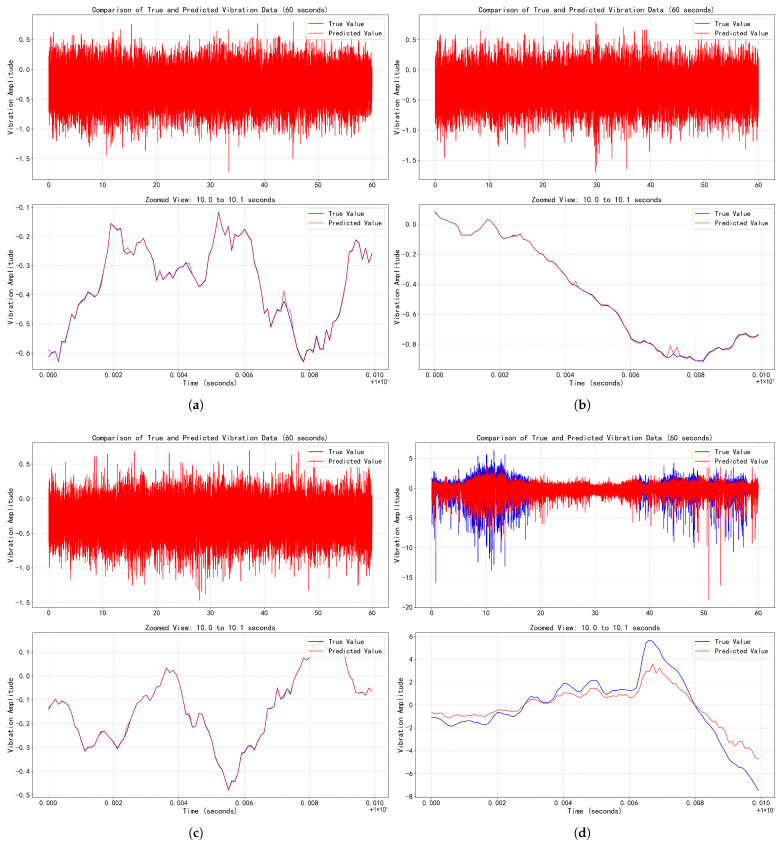
Comparison between the predicted and true curves over a one-min duration within the test set across four consecutive days. (**a**) Comparison between predicted and true values of vibration signals on Day 1 (with partial enlarged view). (**b**) Comparison between predicted and true values of vibration signals on Day 2 (with partial enlarged view). (**c**) Comparison between predicted and true values of vibration signals on Day 3 (with partial enlarged view). (**d**) Comparison between predicted and true values of vibration signals on Day 4 (with partial enlarged view).

Based on the predetermined optimal VMD parameters, the daily collected vibration signals of the EMU motor bearings are decomposed to obtain several IMFs. Subsequently, the proposed deep learning network model is employed to perform time-series forecasting on each IMF component separately. The final predicted vibration signal is reconstructed by superimposing the prediction results of all IMF components. Prediction performance is evaluated by comparing the reconstructed prediction signal with the actual signal. Specifically, for each day’s vibration signal data, an 80%–20% ratio is applied to partition it into training and test sets: the training set is used for model training, while the test set serves to evaluate the prediction accuracy of the model on vibration data under both normal and abnormal operating conditions. Figure 5 presents a comparison between the predicted and true curves over a one-min duration within the test set across four consecutive days. The first three days correspond to vibration signals under normal operating conditions, while the fourth day represents signals under abnormal operating conditions.

To quantitatively evaluate the prediction model performance, three metrics were adopted: RMSE, MAE, and the R^2^, with their calculation formulas defined as follows:

(29) $$\mathrm{RMSE}=\sqrt{\frac{1}{N}\sum_{i=1}^{N}{\left(O_{i}-P_{i}\right)}^{2}}$$

(30) $$\mathrm{MAE}=\frac{1}{N}\sum_{i=1}^{N}\left|O_{i}-P_{i}\right|$$

(31) $$R^{2}=1-\frac{\sum_{i=1}^{N}{\left(O_{i}-P_{i}\right)}^{2}}{\sum_{i=1}^{N}{\left(O_{i}-\bar{O}\right)}^{2}}$$

where $O_{i}$ denotes the *i*-th true value, $P_{i}$ represents the corresponding predicted value, $\bar{O}$ is the mean of true values, and *N* indicates the total number of true values. A smaller RMSE indicates lower deviation between predicted and true values, reflecting higher prediction accuracy. A reduced MAE signifies smaller average prediction error, demonstrating improved model precision. An $R^{2}$ value closer to 1 denotes better goodness of fit. Detailed results are presented in Table 4.

Results from Table 4 and Figure 5 demonstrate that under normal operating conditions (Days 1–3), the predicted signals exhibit high consistency with the true signals (RMSE≤0.006, $R^{2}\geq 0.997$). As shown in Figure 5a–c, the predicted curves are nearly indistinguishable from the true curves (ground truth), including magnified local details, validating the model’s precise capability in capturing healthy states. Under abnormal conditions (Day 4), however, predictive performance degrades significantly (RMSE=0.744, $R^{2}=0.588$). The conspicuous deviation between predicted and true curves in Figure 5d reveals the model’s sensitivity to fault characteristics. These findings confirm the potential of the proposed method for achieving early bearing fault warnings through prediction residuals.

### 4.4. Anomaly Detection in Motor Bearings

#### 4.4.1. Determination of the OC-SVM Hyperparameter ν

The selection of the hyperparameter ν is critical, as it determines the model’s generalization capability and robustness against false alarms. To empirically determine its optimal value, a five-fold time-series cross-validation (CV) was performed exclusively on the normal operational data (Days 1–3). The evaluation was based on two key metrics: (1) the recall of the normal class, which measures the model’s ability to correctly accept normal patterns and thus minimize false alarms; and (2) the standard deviation of the recall across the CV folds, which quantifies the stability and consistency of the model’s performance under different data partitions.

The recall for a single validation fold is calculated as the fraction of normal instances that are correctly identified by the model, defined by:

(32) $$\mathrm{Recall}=\frac{\mathrm{TP}}{\mathrm{TP}+\mathrm{FN}}$$

where TP is the number of normal samples correctly classified as normal, and FN is the number of normal samples incorrectly flagged as anomalies. A higher recall signifies better performance in preserving normal operations.

The standard deviation σ of the recall values across $K_{\mathrm{folds}}$ CV folds is computed to assess consistency:

(33) $$\sigma =\sqrt{\frac{1}{K_{\mathrm{folds}}}\sum_{i=1}^{K}{\left(Recall_{i}-\mu \right)}^{2}}$$

where $K_{\mathrm{folds}}=5$ is the number of folds, $R_{i}$ is the recall measured on the *i*-th validation fold, and μ is the mean recall across all $K_{\mathrm{folds}}$ folds. A lower standard deviation indicates more robust and reliable model performance. A spectrum of candidate ν values was evaluated. The cross-validation results are presented in Table 5.

The results demonstrate that ν=0.05 achieved the dual advantage of the highest mean recall (0.992) and the lowest standard deviation (0.005). This indicates that a model configured with ν=0.05 is not only the most accurate in recognizing normal system behavior but also exhibits the greatest robustness, with minimal performance variance across different data samples. Consequently, ν=0.05 was selected as the optimal value for the final model deployment.

#### 4.4.2. Application of OC-SVM for Anomaly Detection

**Figure 6:**
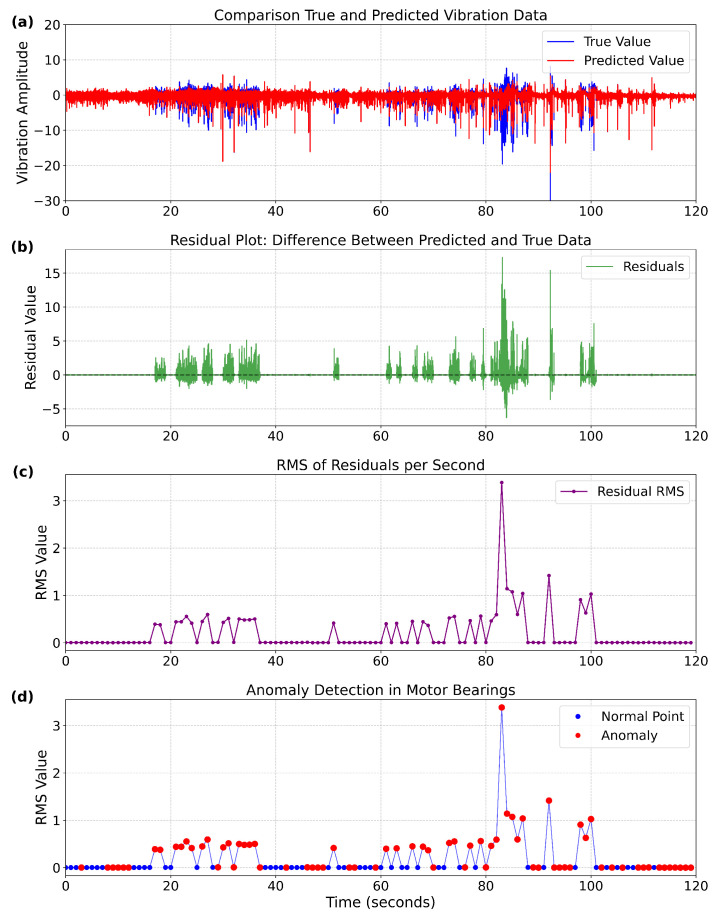
Motor bearing anomaly detection results. (**a**) Comparison of true and predicted vibration data. (**b**) Residual plot: difference between predicted and true data. (**c**) RMS of residuals per second. (**d**) Anomaly detection results for motor bearings in EMU.

Based on the prediction results from the previous section, the residual sequence between the predicted and true values of the motor bearing vibration signal is first calculated. Subsequently, the RMS value of the residuals per second is extracted. Using the residual RMS values obtained from vibration data under normal conditions during the first three days, an OC-SVM model is trained. Finally, the residual RMS values from the fourth day, which contain both normal and abnormal vibration data, are used for testing to achieve bearing anomaly detection. Figure 6 presents the anomaly detection results for a one-min duration during the testing phase.

Figure 6 presents anomaly detection results for a one-min test segment containing both normal and abnormal vibration signals on the fourth day, where subfigure (a) demonstrates close matching between predicted and measured vibration amplitudes of the motor bearing during 0–19 s and 40–60 s (except at 55 s), but reveals significant discrepancies during 19–40 s; subfigure (b) shows substantial residual fluctuations within 20–40 s, indicating the time-series model’s failure to capture abnormal features; subfigure (c) computes the RMS of residuals per second, revealing stable low values in the first 20 s, a transient anomaly (RMS surge to 0.6) during 20–40 s, followed by a return to normal range, while subfigure (d) marks normal (blue) and anomalous (red) time points based on the OC-SVM decision boundary trained on normal data from the first three days. These results collectively demonstrate that OC-SVM training on residual RMS enables efficient detection of abrupt bearing anomalies while confirming the intermittent nature of anomalies in operational scenarios.

### 4.5. Time-Series Prediction Performance Comparison

**Figure 7:**
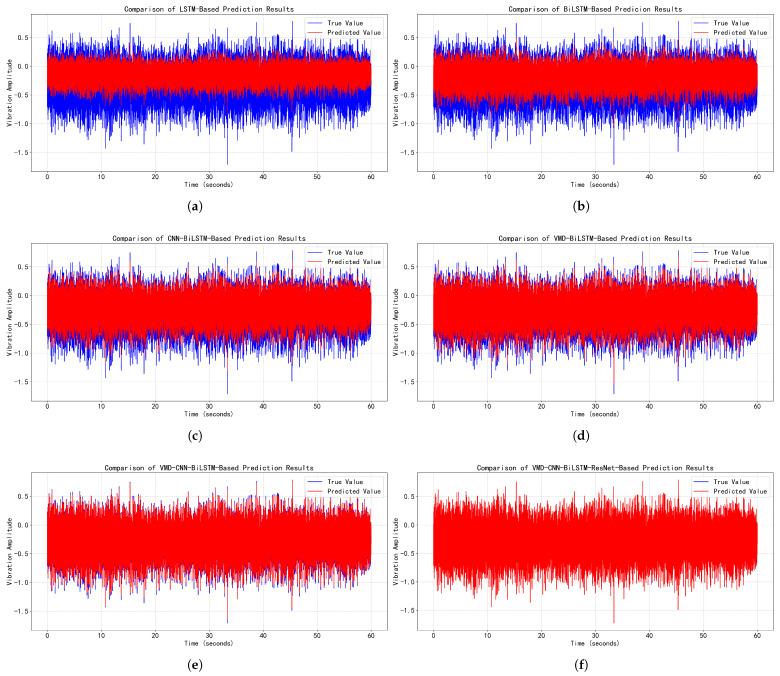
Performance comparison of time-series forecasting models for normal vibration signals of EMU traction motor bearings. (**a**) LSTM-based time-series prediction. (**b**) BiLSTM-based time-series prediction. (**c**) CNN-BiLSTM-based time-series prediction. (**d**) VMD-BiLSTM-based time-series prediction. (**e**) VMD-CNN-BiLSTM-based time-series prediction. (**f**) VMD-CNN-BiLSTM-ResNet-based time-series prediction.

**Figure 8:**
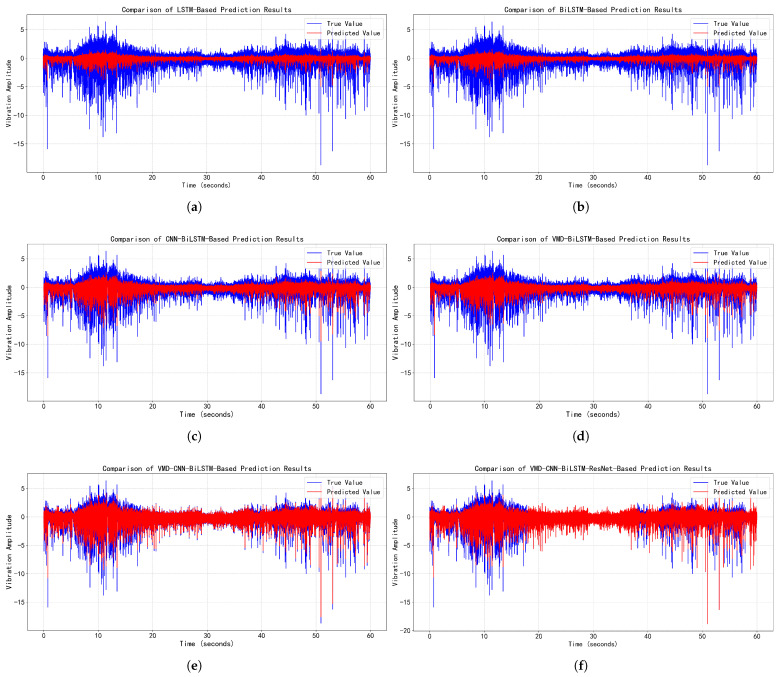
Performance comparison of time-series forecasting models for abnormal vibration signals of EMU traction motor bearings. (**a**) LSTM-based time-series prediction. (**b**) BiLSTM-based time-series prediction. (**c**) CNN-BiLSTM-based time-series prediction. (**d**) VMD-BiLSTM-based time-series prediction. (**e**) VMD-CNN-BiLSTM-based time-series prediction. (**f**) VMD-CNN-BiLSTM-ResNet-based time-series prediction.

To thoroughly validate the predictive performance of the proposed algorithm, time-series forecasting was conducted using both normal vibration signals and data containing anomalous vibrations. Prediction models based on LSTM, BiLSTM, CNN-BiLSTM, VMD-BiLSTM, and VMD-CNN-BiLSTM were employed as comparative methods. The one-min prediction results are contrasted in Figure 7 (normal vibration signals) and Figure 8 (data containing anomalous vibrations).

Simultaneously, RMSE, MAE, and R^2^ were employed to quantitatively evaluate the prediction performance for both normal vibration signals and those containing abnormal vibrations under different time-series forecasting models. Specific results are detailed in Table 6.

As evidenced in Figure 7 and Figure 8 and Table 6, the proposed VMD-CNN-BiLSTM-ResNet model achieves breakthrough performance in time-series forecasting of EMU traction motor bearing vibration signals: it attains near-ideal prediction accuracy for normal vibrations (RMSE=0.005, MAE=0.002, $R^{2}=0.999$), reducing errors by >98% versus baseline LSTM, while maintaining state-of-the-art results for abnormal-vibration-containing signals (RMSE=0.744, MAE=0.410) despite a moderate performance drop in $R^{2}$ (0.588), indicating persistent challenges in modeling complex transient features of anomalies. Progressive architectural optimizations directly drive these gains: VMD preprocessing universally enhances generalization (e.g., VMD-BiLSTM reduces RMSE by 50% over BiLSTM for normal signals), the CNN-BiLSTM hybrid leverages local–global feature synergy to cut abnormal-signal RMSE by 44.7% versus standalone BiLSTM, and ResNet integration mitigates gradient degradation to enable order-of-magnitude improvements (87% RMSE reduction in normal signals for VMD-CNN-BiLSTM-ResNet over VMD-CNN-BiLSTM). Notably, the suboptimal $R^{2}$ for abnormal signals (peak: 0.588) underscores the domain-wide challenge in modeling non-stationary fault signatures, yet this work establishes an actionable framework for high-precision bearing condition forecasting through multistage fusion.

### 4.6. Anomaly Detection Performance Benchmarking

**Figure 9:**
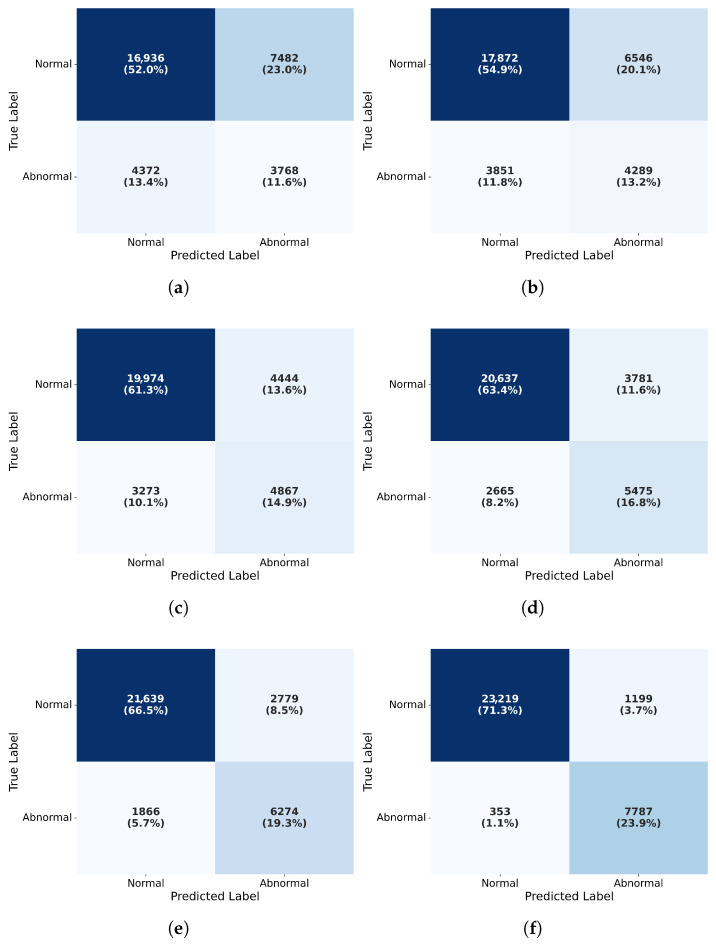
Anomaly detection performance benchmarking. (**a**) Anomaly detection in EMU traction motor bearings based on Isolation Forest. (**b**) Anomaly detection in EMU traction motor bearings based on AE. (**c**) Anomaly detection in EMU traction motor bearings based on CVAE. (**d**) Anomaly detection in EMU traction motor bearings based on CNN-BiLSTM-OC-SVM. (**e**) Anomaly detection in EMU traction motor bearings based on VMD-CNN-BiLSTM-OC-SVM. (**f**) Anomaly detection in EMU traction motor bearings based on VMD-CBR-OCSVM.

To further validate the performance of the proposed anomaly detection algorithm, it is compared against benchmark models including Isolation Forest, AE, Conditional Variational Autoencoder (CVAE), CNN-BiLSTM-OC-SVM, and VMD-CNN-BiLSTM-OC-SVM. The evaluation employs four metrics—accuracy, precision, recall, and F1-score—to quantitatively assess the anomaly detection results for vibration signals in EMU motor bearings. Specifically, vibration samples identified as anomalous are labeled as the positive class, while those identified as normal are labeled as the negative class. Based on this positive/negative class assignment, the evaluation metrics are calculated as follows:

(34) $$\mathrm{Accuracy}=\frac{\mathrm{TP}+\mathrm{TN}}{\mathrm{TP}+\mathrm{TN}+\mathrm{FP}+\mathrm{FN}}$$

(35) $$\mathrm{Precision}=\frac{\mathrm{TP}}{\mathrm{TP}+\mathrm{FP}}$$

(36) $$F1=2\frac{\mathrm{Precision}\cdot \mathrm{Recall}}{\mathrm{Precision}+\mathrm{Recall}}$$

where FP represents samples falsely predicted as positive despite being negative, TN denotes the number of samples correctly predicted as negative that are actually negative; the quantitative evaluation results for anomaly detection in EMU motor bearings are presented in Table 7 and the corresponding confusion matrix (Figure 9).

Based on the performance data in Table 7 and Figure 9 for anomaly detection in EMU traction motor bearings, traditional unsupervised methods (e.g., Isolation Forest) show significant limitations with only 63.6% accuracy, a 0.389 F1-score, and notably low precision (0.335), indicating high false positive rates that fail to meet industrial requirements; generative models (AE, CVAE) improve recall (CVAE: 0.598) but maintain suboptimal precision (<0.53), revealing limited anomaly discrimination specificity. In contrast, deep hybrid models demonstrate substantial superiority: the CNN-BiLSTM-OC-SVM framework integrating spatio-temporal feature extraction with one-class SVM elevates the F1-score to 0.630 (14.7% relative gain), validating bidirectional LSTM’s efficacy in capturing vibration temporal features; the VMD-CNN-BiLSTM-OC-SVM model further optimizes feature quality through variational mode decomposition (VMD) preprocessing, achieving 0.693 precision and 0.771 recall, highlighting VMD’s critical role in noise suppression and fault feature enhancement. Ultimately, the proposed VMD-CBR-OCSVM model with residual network integration achieves breakthrough performance: near-perfect recall (0.957) minimizes missed detections to ensure safety-critical system reliability; balanced high precision (0.867) and F1-score (0.909) represent a 0.279 absolute F1 improvement (44.4% relative gain) over the best baseline; and 95.2% accuracy robustly validates the multi-stage deep feature-fusion architecture’s strong adaptability to complex operating conditions.

## 5. Conclusions and Future Work

This paper proposed an integrated vibration anomaly detection method for motor bearings in EMU by fusing VMD with deep learning. The main findings and contributions of this study are three-fold:

First, a power-spectrum-guided adaptive VMD parameter optimization mechanism was established, which effectively overcame the mode-mixing problem and enabled adaptive decomposition of highly non-stationary vibration signals.

Second, a hybrid CNN-BiLSTM-ResNet model was developed, which achieved near-perfect prediction performance ($R^{2}\geq 0.997$, RMSE≤0.006) on normal operational data by leveraging multi-scale feature extraction and temporal dependency modeling.

Third, an unsupervised anomaly detection framework based on prediction-error RMS and OC-SVM was constructed. Most significantly, this framework demonstrated superior performance on real-world operational data, achieving an accuracy of 95.2% and an F1-score of 0.909 in detecting anomalous states. This represents a substantial improvement (e.g., a 44.4% relative increase in F1-score) over conventional methods like Isolation Forest, highlighting its practical efficacy. Despite the demonstrated effectiveness of the RMS feature for trend-based anomaly detection in this non-real-time monitoring context, it is noteworthy that feature engineering offers avenues for further enhancement. The RMS value was chosen for its robustness in capturing energy-based deviations indicative of gradual degradation. However, other feature domains, such as kurtosis for impulsivity or entropy for signal complexity, could offer complementary benefits for detecting a wider variety of fault types, particularly incipient localized faults.

The significance of these findings lies in providing a reliable, annotation-free, technical solution for early fault detection and warning in EMU motor bearings under complex operational environments. This approach effectively addresses the industrial challenge of scarce fault data and enables trend analysis using readily available normal vibration data.

Future research will focus on two primary directions to translate these findings into practical applications:

First, methodological advancements through multi-source data fusion (e.g., integrating temperature and rotational speed) employing ensemble and transfer learning to further enhance detection precision and robustness. Building on the feature discussion above, a core aspect will be the exploration of multi-feature fusion strategies within this framework, combining time-domain, frequency-domain, and information-theoretic features to further improve detection sensitivity, specificity, and overall robustness.

Second, we will focus on the development and deployment of a real-time online monitoring and early-warning system for EMU motor bearing health management. As a direct application of this framework, the system will incorporate multi-dimensional features such as RMS, kurtosis, and entropy to enhance the sensitivity and reliability of condition monitoring, thereby ensuring safe and stable train operations.

## Figures and Tables

**Table 1:** Detailed architecture and hyperparameters of the CNN-BiLSTM-ResNet prediction model.

Component	Layer Type	Kernel Size	Stride	Padding	Channels
Encoder	Conv 1	3 × 1	1	1	64
	Conv 2	3 × 1	1	1	128
	Conv 3	3 × 1	1	1	256
	Conv 4	3 × 1	1	1	512
	MaxPool	4 × 1	2	1	-
Sequence Modeling	BiLSTM Layer 1	-	-	-	256
	BiLSTM Layer 2	-	-	-	128
Decoder	Upsample	-	-	-	-
	Conv 1	3 × 1	1	1	256
	Conv 2	3 × 1	1	1	128
	Conv 3	3 × 1	1	1	64
	Conv 4	3 × 1	1	1	1
Training	Optimizer	Adam (lr = 0.00001)			
	Batch Size	64			
	Epochs	100			
	Loss Function	Mean Squared Error			

**Table 2:** Initial selection range of VMD decomposition parameter K.

Date	Initial Selection Range of K
Day 1	[8, 12]
Day 2	[7, 11]
Day 3	[9, 13]
Day 4	[4, 8]

**Table 3:** Optimal decomposition parameters of motor bearing vibration signals for different dates.

Date	$K_{p}$	$\alpha_{p}$
Day 1	9	600
Day 2	9	600
Day 3	11	500
Day 4	5	1000

**Table 4:** Comparison of prediction performance metrics for vibration signals under normal and abnormal conditions.

Date	RMSE	MAE	R^2^
Day 1	0.005	0.002	0.999
Day 2	0.005	0.002	0.999
Day 3	0.006	0.002	0.997
Day 4	0.744	0.410	0.588

**Table 5:** Cross-validation results for hyperparameter ν selection.

ν Value	Mean Recall (μ)	Std. Deviation (σ)
0.01	0.975	0.018
0.03	0.988	0.008
0.05	0.992	0.005
0.07	0.990	0.012
0.10	0.985	0.015

**Table 6:** Prediction performance comparison of normal vs. abnormal vibration-containing signals under different time-series forecasting models.

Prediction Model	Normal Vibration Signals			Abnormal Vibration-Containing Signals		
	RMSE	MAE	R^2^	RMSE	MAE	R^2^
LSTM	0.257	0.210	−0.110	5.979	8.149	0.139
BiLSTM	0.205	0.189	0.312	2.354	5.271	0.205
CNN-BiLSTM	0.141	0.106	0.697	1.302	1.354	0.455
VMD-BiLSTM	0.103	0.093	0.798	0.874	0.837	0.432
VMD-CNN-BiLSTM	0.039	0.026	0.974	0.796	0.503	0.627
VMD-CNN-BiLSTM-ResNet	0.005	0.002	0.999	0.744	0.410	0.588

**Table 7:** Evaluation results of motor bearing anomaly detection.

Prediction Model	Accuracy	Precision	Recall	F1
Isolation Forest	0.636	0.335	0.463	0.389
AE	0.681	0.396	0.527	0.452
CVAE	0.763	0.523	0.598	0.558
CNN-BiLSTM-OC-SVM	0.802	0.592	0.673	0.630
VMD-CNN-BiLSTM-OC-SVM	0.857	0.693	0.771	0.730
VMD-CBR-OCSVM	0.952	0.867	0.957	0.909

## Data Availability

The datasets supporting this study are available from the corresponding author upon reasonable request. Due to confidentiality requirements from the China Academy of Railway Sciences (CARS), public availability is restricted as the data contain technical parameters of high-speed EMU systems. Researchers interested in accessing the data may contact the corresponding author to discuss potential collaboration pathways.
